# A Third-generation Lentiviral Vector System for Flow Virometry-based Analysis of HIV-1 Env and Other Viral Envelope Proteins

**DOI:** 10.64898/2026.10.11.755930

**Published:** 2026-10-12

**Authors:** Paula I. Busskamp Castro, Janine Parreuter, Charlotte Brands, Ana-Maria Perkovic, Benton van Bilsen, Bernd Giebel, Jens Bohne, Tobias Tertel, Jamal Ghanam, Basant K. Thakur, Christina B. Karsten

## Abstract

Individual human immunodeficiency virus (HIV) particles can differ in their envelope protein (Env) despite being genetically identical. This non-genetic diversity can influence antibody recognition and has been implicated in the formation of persistent virus fractions that escape complete inhibition by broadly neutralizing antibodies. Investigating non-genetic diversity in HIV and other viruses requires approaches capable of characterizing antibody binding to individual viral particles. Flow virometry provides a promising tool for single-particle analysis, but a system combining high biosafety with specific particle labeling and versatility is lacking. Here, we developed a third-generation lentiviral vector system specifically designed for flow virometry.

Fluorescent proteins were incorporated into the HIV-1 Gag polyprotein between the matrix- and capsid-encoding regions, flanked by viral protease cleavage sites, creating the packaging constructs pLentiFlow-eGFP and pLentiFlow-mCherry. Lentiviral particles (LVPs) based on pLentiFlow vectors were fluorescent and retained infectivity comparable to LVPs generated with the parental vector system despite reduced particle production. We optimized LVP detection on a CytoFLEX nano and defined a linear p24-based concentration range for reliable single-particle analysis. Using complementary controls, we demonstrated that background from the assessed sources accounted for no more than approximately 3% of events within a broad eGFP+ gate and confirmed that the detected fluorescent particles were predominantly membrane-enclosed. As a proof of concept, LVPs were pseudotyped with vesicular stomatitis virus glycoprotein, severe acute respiratory syndrome coronavirus 2 spike, or HIV-1 Env. Envelope-specific antibody binding was detected using 8G5F11, CR3022 or PGT128, respectively, demonstrating that different viral surface proteins can be analyzed on a common particle backbone.

Together, these results establish a third-generation lentiviral vector system for flow virometry that combines intrinsic particle labeling with high biosafety and flexible pseudotyping. The system provides a platform for investigating envelope protein-associated non-genetic diversity and its consequences for antibody recognition in HIV and other viruses.

**Author summary:**

Recent studies suggest that non-genetic diversity in the envelope protein of the human immunodeficiency virus (HIV) influences antibody recognition, a phenomenon potentially relevant to other viruses as well. Understanding such non-genetic differences may aid the development of antibody-based vaccines and therapies. To assess envelope protein-associated non-genetic diversity within viral swarms, methods are needed to analyze individual viruses. Flow virometry is a promising approach but current methods face challenges in combining single-particle detection by flow cytometry with high biosafety and versatility.

Here, we present a third-generation lentiviral vector system that generates fluorescent lentiviral particles (LVPs) for direct detection. These LVPs are produced from a reduced and split HIV genome and cannot produce infectious virus after infecting a cell, providing a high level of biosafety. The incorporated viral envelope proteins can be exchanged, allowing the study of HIV and other viruses. We characterize the LVPs, optimize their detection, establish conditions for reliable analysis of individual particles, and demonstrate the versatility of the system by detecting antibody binding to envelope proteins from HIV and two other viruses. By combining biosafety and versatility, this system broadens access to flow virometry and enables the study of non-genetic differences within swarms of HIV and other viruses.

